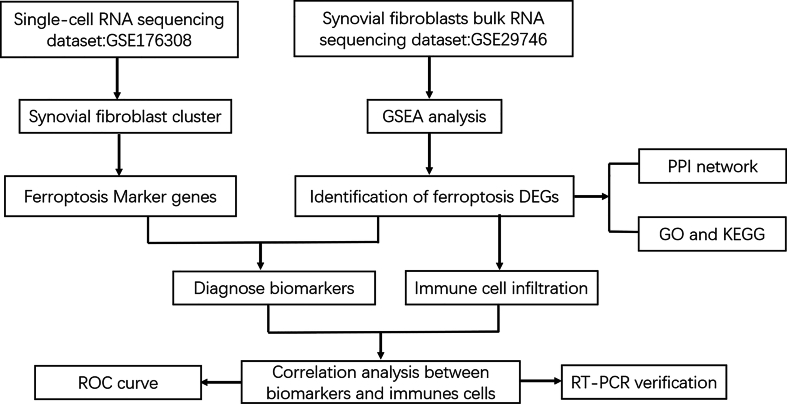# Bulk RNA sequencing combined with single-cell RNA sequencing analysis revealed the ferroptosis immune target of osteoarthritis synovial fibroblasts

**DOI:** 10.1016/j.gendis.2025.101587

**Published:** 2025-03-04

**Authors:** Qian Wu, Liang Zhou, Yuan Xue, Jiaqian Wang

**Affiliations:** a Department of Orthopedic Surgery, The First Affiliated Hospital, Suzhou Medical College, Soochow University, Suzhou, Jiangsu 215006, China; b Department of Orthopedics, Huai'an Hospital Affiliated to Yangzhou University, The Fifth People's Hospital of Huai'an, Huai'an, Jiangsu 223399, China; c Department of Orthopedics, Wuxi Ninth People's Hospital Affiliated to Soochow University, Wuxi, Jiangsu 214026, China; d Department of Orthopaedic Surgery, Zhongshan Hospital, Fudan University, Shanghai 200032, China

Osteoarthritis (OA) is the most common joint disease in elderly patients. Its main pathological change is articular cartilage degeneration, accompanied by synovial inflammation and changes in subchondral bone structure, resulting in pain and limited mobility. However, previous studies on OA mainly focused on the dysfunction of cartilage and chondrocytes, and the synovium and other joint structures have not received enough attention. Synovium has shown inflammatory changes to varying degrees before the morphological changes of articular cartilage occur in the early stage.^1^ Synovial fibroblasts are the most important cell type in the synovium of arthritic hyperplasia. A large number of studies have shown that fibroblasts in synovium participate in the activities of arthritis, but the specific molecular mechanism is still unclear.^2^ Ferroptosis refers to a new type of cell death caused by the abnormal accumulation of iron-dependent reactive oxygen species, which leads to the imbalance of oxidation and reduction. Zhu et al found that by activating the ferroptosis pathway, ferroptosis can reduce the number of synovial fibroblasts in the mouse arthritis model, thereby inhibiting the damage to articular cartilage and delaying the progress of arthritis.^3^ Therefore, the ferroptosis pathway targeting synovial fibroblasts can provide a new therapeutic strategy for OA and better study its molecular mechanism.

Bulk RNA sequencing provides a tissue-wide transcription landscape. Single-cell sequencing reveals the gene expression status of a single cell. In our study, we combined two sequencing techniques to more comprehensively explore the role of synovial fibroblasts in OA. First, we searched the Gene Expression Omnibus (GEO) database with the keywords of “osteoarthritis” and “synovial fibroblasts”. We obtained the bulk RNA sequencing dataset (GSE29746) of synovial fibroblasts from patients with OA and normal people, and the single-cell RNA sequencing dataset (GSE176308) of synovial fibroblasts from patients with OA. We preprocessed the dataset GSE29746, which includes 11 normal control samples and 11 synovial fibroblast samples from patients with OA. After downloading the raw data, we deleted the rows and columns with a missing value ratio greater than 50%, and further used the R package “impute” to complete the missing values. Gene set enrichment analysis (GSEA) was used to compare the effect of synergistic changes of genes in the gene set of OA and normal synovial fibroblasts on phenotypic changes. GSEA detects gene sets rather than individual gene expression changes, so it can include these subtle expression changes. We found that the biological process of synovial fibroblasts in OA patients mainly focused on fatty acid oxidation, cell apoptosis process, and T cell and fiber cell differentiation (Fig. 1A). Molecular functions are thioredoxin peroxidase, glutathione conjugate transporter, superoxide generating, and ferric ion binding (Fig. S1). Apoptosis, oxidative phosphorylation, and fatty acid and glutathione metabolism are signaling pathways related to OA (Fig. 1B).

**Figure 1:**
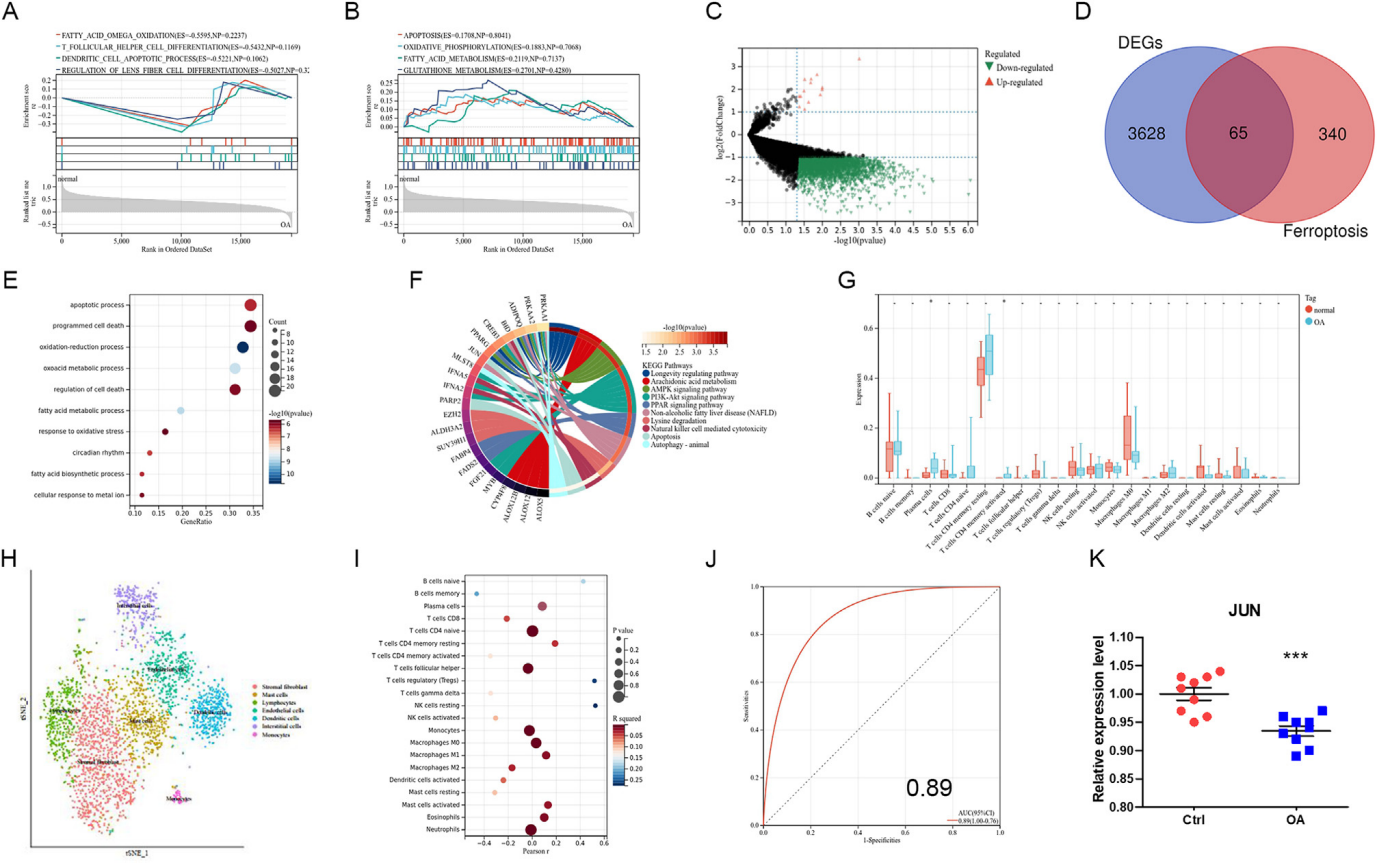
Bulk RNA sequencing combined with single-cell RNA sequencing analysis revealed the ferroptosis immune target of osteoarthritis (OA) synovial fibroblasts. **(A)** GSEA analysis of GO biological process differences between OA and normal synovial fibroblasts. **(B)** GSEA analysis of KEGG pathway. **(C)** The volcano map showing differentially expressed genes (DEGs) between OA and normal synovial fibroblasts. **(D)** Venn diagrams extracted DEGs related to ferroptosis. **(E)** GO biological process of DEGs related to ferroptosis. **(F)** KEGG pathway of DEGs related to ferroptosis. **(G)** The violin diagram showing the difference in immune cells between OA and control. **(H)** t-SNE analysis divides the cells into seven different clusters. The cell type was determined according to marker genes in each cluster. **(I)** Correlation between JUN and immune cells. **(J)** Operating characteristic curve of JUN. **(K)** Quantitative PCR validation of JUN.

Based on the GSEA results of synovial fibroblasts, we speculated that ferroptosis plays an important role in OA. Differentially expressed genes (DEGs) of OA patients and normal synovial fibroblasts were identified by the “limma” package. The volcanic map shows the difference level of DEGs, and the false discovery rate <0.05 along with |log2 fold change| >1 was considered statistically significant. 14 up-regulated DEGs and 3679 down-regulated DEGs were screened out in the normal and OA groups (Fig. 1C). 405 ferroptosis-related genes were downloaded from the FerrDb database, and the DEGs related to ferroptosis were extracted through the intersection of Venn diagrams. 65 ferroptosis-related DEGs were screened out (Fig. 1D). Gene Ontology (GO) and Kyoto Encyclopedia of Genes and Genomes (KEGG) analysis of DEGs were performed by R package “clusterProfiler”. The biological process of ferroptosis-related DEGs mainly focused on apoptosis, cell death, oxidation-reduction, and fatty acid metabolic process (Fig. 1E). Longevity regulating, arachidonic acid metabolism, and AMPK signaling pathway are the main enriched KEGG pathway (Fig. 1F).

In addition, we also found that synovial fibroblasts of OA were enriched in T cell- and dendritic cell-related functional pathways (Fig. 1A). Therefore, we further used the CIBERSORT algorithm to compare the infiltration of immune cells in OA and normal synovial fibroblasts. The violin diagram showed that the proportion of plasma cells and activated memory CD4 T cells in synovial fibroblasts of OA patients was significantly higher (Fig. 1G). Naïve B cells, resting memory CD4 T cells, and M0 macrophages were the main infiltrating immune cells in the synovium fibroblasts (Fig. S2).

To further verify the relationship between ferroptosis-related genes and immune cells in synovial fibroblasts, we analyzed the results of single-cell RNA sequencing. The single-cell RNA sequence dataset contains 29 early-stage and 22 end-stage OA synovial fibroblast samples. t-SNE analysis visualized seven clusters (Fig. 1H). According to the top ten marker genes of each cluster, the cell population diagram was manually annotated: cluster 0 (stromal fibroblast), cluster 1 (mast cells), cluster 2 (lymphocytes), cluster 3 (endothelial cells), cluster 4 (dendritic cells), cluster 5 (interstitial cells), and cluster 6 (monocytes). The common genes in ferroptosis-related DEGs and single-cell marker genes were used as diagnostic markers. The overlap included three genes, JUN, SLC40A1, and SNCA (Fig. S3). JUN was positively correlated with naïve B cells (*r* = 0.4251, *P* = 0.0122), regulatory T cells (*r* = 0.5183, *P* = 0.0017), resting natural killer cells (*r* = 0.5259, *P* = 0.0014), and negatively correlated with activated memory CD4 T cells (*r* = −0.3487, *P* = 0.0433), gamma delta T cells (*r* = −0.3463, *P* = 0.0448) (Fig. 1I). The diagnostic value of biomarkers was judged by the operating characteristic curve of the subjects. The area under the curve = 0.89, indicating that the diagnostic value of JUN was good (Fig. 1J). Quantitative reverse transcription PCR further verified the validity of the immune diagnostic marker JUN (Fig. 1K).

For a long time, OA has been considered a joint disease caused by articular cartilage degeneration. With the deepening understanding of OA inflammation, the position of synovium in OA has been enhanced. According to bulk RNA sequencing results, 65 ferroptosis-related genes in OA synovial fibroblasts were down-regulated compared with normal synovial fibroblasts. Early studies confirmed that mesenchymal cells such as fibroblasts were sensitive to ferroptosis. Ferroptosis inducers reduced the number of synovial fibroblasts and alleviated synovial inflammation.^4^ AMPK, P53, and glutathione metabolism signal pathways are the main OA ferroptosis enrichment pathways. These pathways ultimately affect the activity of glutathione peroxidase directly or indirectly, reducing the antioxidant capacity of cells, and leading to the increase of reactive oxygen species and the occurrence of ferroptosis. Induced ferroptosis may be a target for the treatment of OA synovitis. Combined with bulk RNA sequencing and single-cell RNA sequencing results, lymphocytes are the most important immune cells in OA synovial fibroblasts. JNK is a classical signal pathway involved in the progression of OA and plays an important role in cartilage destruction. The activation of JNK leads to the phosphorylation of c-Jun, which leads to the reduction of proteoglycan synthesis and the increase of matrix metalloproteinase 13 (MMP-13) production. Glutamine can inhibit JNK activity and play an anti-inflammatory and cartilage-protective role in OA.^5^ JNK signaling pathway can also regulate the differentiation of CD4^+^ T cells and participate in the disease activity and treatment of arthritis. Based on the above results, JUN may regulate the ferroptosis of OA synovial fibroblasts by targeting lymphocytes and may be the immune target of OA synovial fibroblasts after verification.

## CRediT authorship contribution statement

**Qian Wu:** Data curation, Investigation, Visualization, Writing – review & editing. **Liang Zhou:** Funding acquisition, Resources, Supervision, Writing – review & editing. **Yuan Xue:** Data curation, Methodology, Validation, Writing – review & editing. **Jiaqian Wang:** Conceptualization, Project administration, Software, Writing – original draft, Writing – review & editing.

## Ethics declaration

This study was approved by the Fifth People's Hospital of Huai'an research ethics committee (Ethics approval name: Differential gene expression in synovial tissue of knee joint; Ethics approval date: November 22, 2023). Informed consent was obtained from all individual participants included in the study.

## Data availability

The data used to support the findings of this study are included in the article.

## Conflict of interests

The authors declared no conflict of interests.

## Figures and Tables

**Fig. S1:**
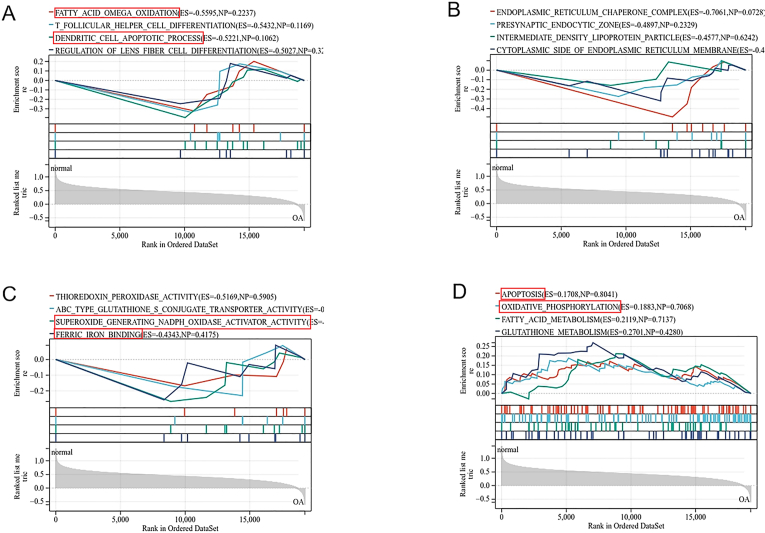


**Fig. S2:**
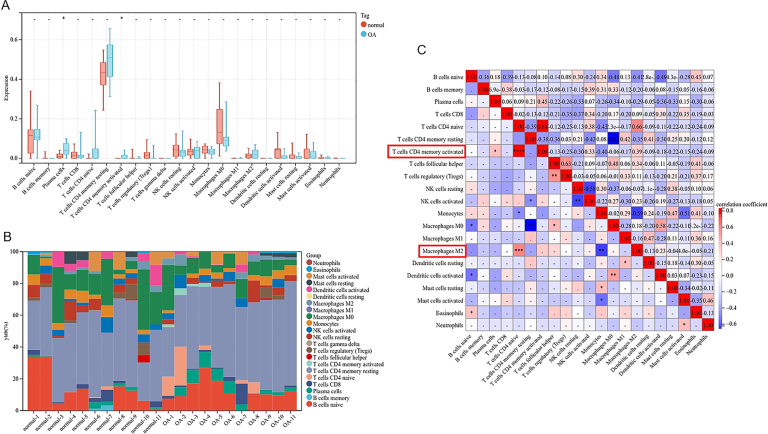


**Fig. S3:**
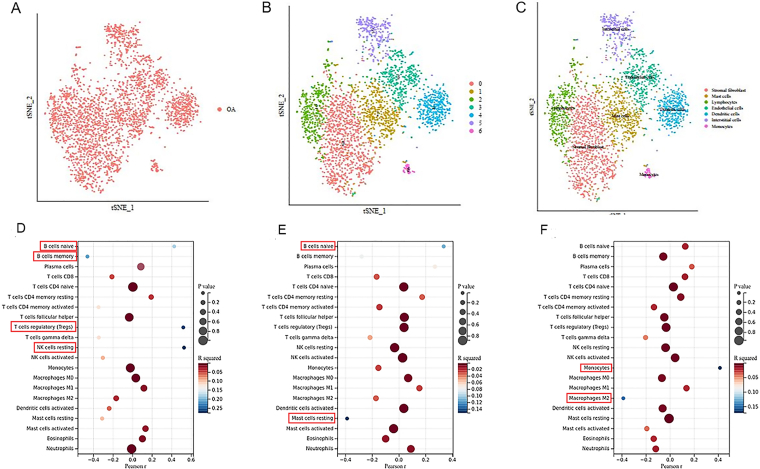


**Fig. S4:**
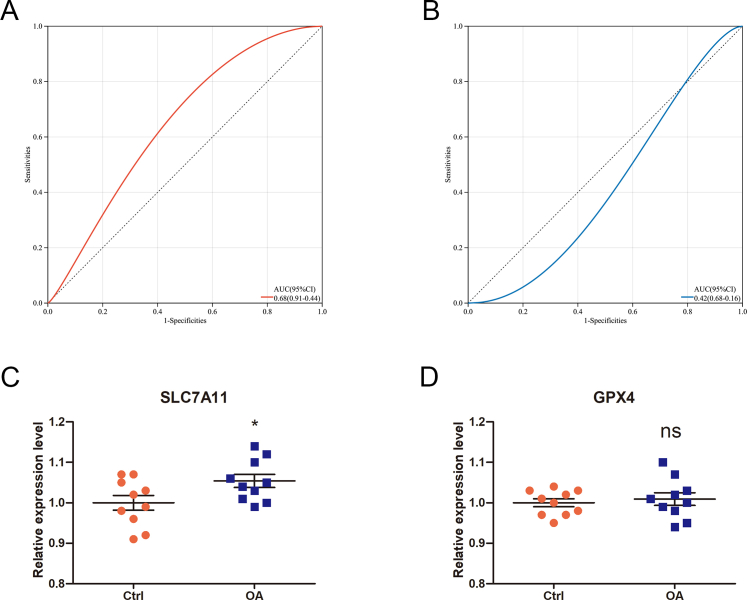


**Fig. S5:**