# Supplementary material for: Bulk RNA sequencing combined with single-cell RNA sequencing analysis revealed the ferroptosis immune target of osteoarthritis synovial fibroblasts
Source: Genes Dis. 2025 Mar 4;12(6):101587. doi: 10.1016/j.gendis.2025.101587 (PMC12272416; doi:10.1016/j.gendis.2025.101587)
Supplement: Multimedia component 1 [file mmc1.docx]

**Materials and methods**

**Datasets Collection**

The flowchart of this study is shown in **Supplementary Figure 4**. We search the Gene Expression Omnibus (GEO) database with the keywords of "osteoarthritis" and "synovial fibroblasts". The studies included in the analysis met the following criteria: (1) Sequencing research with complete data; (2) Homo sapiens gene expression profile of osteoarthritis; (3) Tissue samples of synovial fibroblasts from patients' knee joints. We obtained the comparative dataset (GSE29746) of synovial fibroblasts from patients with OA and normal people, and the single-cell RNA sequencing dataset (GSE176308) of synovial fibroblasts from patients with OA.

**Data preprocessing and** **Gene set enrichment analysis**

We first preprocess the dataset GSE29746, which includes 11 normal control samples and 11 synovial fibroblast samples from patients with OA. After downloading the raw data, we deleted the rows and columns with the missing value ratio greater than 50%, and further used the R package " impute" to complete the missing values. Gene set enrichment analysis (GSEA) was used to compare the effect of synergistic changes of genes in the gene set of osteoarthritis and normal synovial fibroblasts on phenotypic changes. For GSEA analysis, we use the GO annotation of genes in R package “org.Hs.eg.db” as the background, map genes to the background set, and use R package “clusterProfiler” for enrichment analysis to obtain the results of gene set enrichment. Four significantly different Gene Ontology (GO) biological processes, cellular components, molecular functions and Kyoto Encyclopedia of Genes and Genomes (KEGG) pathways were screened.

**Screening and functional enrichment analysis of differential expressed ferroptosis related genes**

Differential expression genes (DEG) of OA patients and normal synovial fibroblasts were identified by "limma" package. The volcanic map shows the difference level of DEGs, and the false discovery rate (FDR) <0.05 and | log2FC | >1 was considered to be statistically significant. The volcanic map can visually observe the overall distribution of expression multiples and the overall distribution of significant differences.405 ferroptosis related genes were downloaded from the FerrDb database, and the DEGs related to ferroptosis was extracted through the intersection of Venn diagrams. The heatmap shows the expression level of ferroptosis related DEGs in each sample. GO and KEGG analysis of DEGs are performed by R package "clusterProfiler". Ten significantly different GO biological processes, cellular components, molecular functions and KEGG pathways were screened by *p* value <0.05.

**Evaluation of immune cells infiltration**

CIBERSORT algorithm was used to compare the infiltration of immune cells in OA and normal synovial fibroblasts. The algorithm uses LM22 characteristic matrix to define 22 kinds of infiltrating immune cell components, and only retains the data with *p* value <0.05.

**Single-cell RNA sequencing data processing**

The single-cell RNA sequence dataset contains two OA synovial fibroblast samples. Seurat package filtered out cells with <1000 genes,> 6000 genes or >10% mitochondrial genes. For the filtered data, according to the feature subsets of hypervariable genes, the batch removal analysis is conducted according to these feature sets. After batch removal, select the gene with the largest difference in expression between cells (HVGs). Then, principal component analysis (PCA) was used to determine the highly variable gene population and *p* value distribution. Finally, t-SNE analysis divides the cells into 7 different clusters and determines the marker gene in each cluster.

**Predictive biomarkers and correlation analysis**

The common genes in ferroptosis related DEGs and single cell marker genes are used as diagnostic markers. Spearman’s rank was used to analyze the correlation between the identified diagnostic biomarkers and infiltrating immune cells, bubble chart was used for visualization. The diagnostic value of biomarkers was judged by the operating characteristic curve (ROC) of the subjects. The area under the curve (AUC) was >0.8, indicating that the diagnostic value was good.

**qRT‐PCR and statistical analysis**

All R packages are implemented through RStudio (v4.1.3). This study was approved by local ethics committee. All methods were performed in accordance with the relevant guidelines and regulations. Synovial tissue was obtained from 10 OA patients who underwent total knee arthroplasty, and the synovium of 10 normal patients who underwent femoral amputation due to trauma was used as control. The age and gender of the patients are shown in **Supplementary Table 1**. Total RNA was extracted using Trizol reagent (Beyotime, China) and reverse transcribed. QRT-PCR was performed on CFX connect real-time PCR detection system (Bio-rad, USA). The 2^-ΔΔCt^ methods were used to calculate the expression of three diagnostic genes[19]. GAPDH normalized the expression level of target genes. All experiments were performed independently in triplicate. Specific primer sequences are shown in **Supplementary Table 2**.
