# Supplementary material for: Bulk RNA sequencing combined with single-cell RNA sequencing analysis revealed the ferroptosis immune target of osteoarthritis synovial fibroblasts
Source: Genes Dis. 2025 Mar 4;12(6):101587. doi: 10.1016/j.gendis.2025.101587 (PMC12272416; doi:10.1016/j.gendis.2025.101587)
Supplement: Multimedia component 2 [file mmc2.docx]

**Figure legends：**

**Figure S1.** Compare the functional differences between OA and normal synovial fibroblasts, we analyzed their gene expression profiles dataset GSE29746 using GSEA. (A) GO biological process. (B) GO molecular function. (C) GO cellular component. (D) KEGG pathway.

**Figure S2.** Immune cell infiltration of OA and control synovial fibroblasts. (A) Violin diagram shows the difference of immune cells between OA and control. (B) Histogram shows the composition of 22 immune cells in OA and control. (C) Correlation heatmap shows the relationship between immune cells. (The statistically significant difference was * p<0.05, ** p<0.01, *** p<0.001)

**Figure S3.** Marker genes of each cluster and correlation between ferroptosis biomarkers and immune cells. (A) t-SNE analysis of synovial fibroblasts. (B) t-SNE analysis divides the cells into 7 different clusters. (C) Determine cell type according to marker genes in each cluster. (D) Correlation between JUN and immune cells. (E) Correlation between SLC40A1 and immune cells. (f) Correlation between SNCA and immune cells.

**Figure S4. Key molecules in the ferroptosis signaling pathway regulated by JUN in synovial tissue samples from OA patients. (A) ROC curve of SLC7A11. (B) ROC curve of GPX4.** **(C) qPCR validation of SLC7A11. (D) qPCR validation of GPX4. (The statistically significant difference was * p<0.05, ** p<0.01, *** p<0.001)**

**Figure S5.** Flowchart of screening ferroptosis immune targets of OA synovial fibroblasts by combining bulk RNA sequencing and single-cell RNA sequencing.

**Supplementary Table 1. Clinical characteristics of 10 OA patients and 10 normal controls**

| **Patients** | **Tissue** | **Group** | **Gender** | **Age** |
| --- | --- | --- | --- | --- |
| **1** | **synovium** | **OA** | **F** | **57** |
| **2** | **synovium** | **OA** | **M** | **66** |
| **3** | **synovium** | **OA** | **F** | **64** |
| **4** | **synovium** | **OA** | **F** | **63** |
| **5** | **synovium** | **OA** | **F** | **67** |
| **6** | **synovium** | **OA** | **M** | **65** |
| **7** | **synovium** | **OA** | **F** | **70** |
| **8** | **synovium** | **OA** | **M** | **66** |
| **9** | **synovium** | **OA** | **M** | **62** |
| **10** | **synovium** | **OA** | **M** | **69** |
| **1** | **synovium** | **Control** | **F** | **59** |
| **2** | **synovium** | **Control** | **F** | **64** |
| **3** | **synovium** | **Control** | **F** | **67** |
| **4** | **synovium** | **Control** | **M** | **69** |
| **5** | **synovium** | **Control** | **M** | **67** |
| **6** | **synovium** | **Control** | **F** | **62** |
| **7** | **synovium** | **Control** | **M** | **64** |
| **8** | **synovium** | **Control** | **M** | **68** |
| **9** | **synovium** | **Control** | **F** | **66** |
| **10** | **synovium** | **Control** | **M** | **61** |

**Supplementary Table 2. Primers of ferroptosis target genes for qPCR**

| **Genes** | **F Sequences (5’— 3’)** | **R Sequences (5’— 3’)** |
| --- | --- | --- |
| **GAPDH** | **GTCTCCTCTGACTTCAACAGCG** | **ACCACCCTGTTGCTGTAGCCAA** |
| **JUN** | **GAGCTGGAGCGCCTGATAAT** | **CCCTCCTGCTCATCTGTCAC** |
| **SLC7A11** | **TCTCCAAAGGAGGTTACCTGC** | **AGACTCCCCTCAGTAAAGTGAC** |
| **GPX4** | **GAAGTAAACTACACTCAGCTC** | **CTCTTTGATCTCTTCGTTACTC** |

**Supplementary Table 3. Marker genes for each cluster of synovial fibroblasts**

| Gene | p_val | avg_log2FC | pct.1 | pct.2 | p_val_adj | cluster |
| --- | --- | --- | --- | --- | --- | --- |
| SERPINF1 | 8.65E-194 | 1.153401 | 0.992 | 0.727 | 1.73E-190 | 0 |
| COL6A3 | 4.55E-189 | 0.968215 | 0.999 | 0.923 | 9.11E-186 | 0 |
| CST3 | 3.42E-170 | 0.773305 | 1 | 0.998 | 6.84E-167 | 0 |
| MMP2 | 4.31E-169 | 0.813764 | 1 | 0.994 | 8.62E-166 | 0 |
| WISP2 | 2.10E-146 | 1.033856 | 0.999 | 0.966 | 4.20E-143 | 0 |
| FOS | 5.79E-143 | 1.266249 | 0.972 | 0.687 | 1.16E-139 | 0 |
| CADM1 | 8.04E-131 | 0.594816 | 0.861 | 0.409 | 1.61E-127 | 0 |
| COL6A2 | 4.58E-130 | 0.692743 | 0.998 | 0.964 | 9.17E-127 | 0 |
| CLU | 7.04E-121 | 0.920341 | 0.973 | 0.755 | 1.41E-117 | 0 |
| \| SLC40A1 \| 1.87E-110 \| 0.324787 \| 0.773 \| 0.38 \| 3.74E-107 \| 0 \| \| --- \| --- \| --- \| --- \| --- \| --- \| --- \| | 3.44E-116 | 0.763594 | 0.972 | 0.727 | 6.88E-113 | 0 |
|  |  |  |  |  |  |  |
| CXCL12 | 5.49E-100 | 1.339423 | 0.992 | 0.852 | 1.10E-96 | 1 |
| LINC01423 | 4.86E-98 | 1.750005 | 0.846 | 0.456 | 9.72E-95 | 1 |
| TMEM176A | 7.07E-68 | 0.945479 | 0.689 | 0.261 | 1.41E-64 | 1 |
| RPS4Y11 | 1.76E-66 | 0.809897 | 0.961 | 0.677 | 3.52E-63 | 1 |
| PTGS2 | 8.29E-61 | 0.843516 | 0.741 | 0.489 | 1.66E-57 | 1 |
| FAM118A | 1.50E-55 | 0.403224 | 0.763 | 0.489 | 3.00E-52 | 1 |
| IGFBP4 | 2.08E-53 | 0.634958 | 1 | 0.996 | 4.16E-50 | 1 |
| LIF | 8.73E-53 | 0.838429 | 0.868 | 0.711 | 1.75E-49 | 1 |
| EGR3 | 4.22E-51 | 0.684996 | 0.967 | 0.878 | 8.44E-48 | 1 |
| TIPARP | 4.53E-50 | 0.594782 | 0.819 | 0.636 | 9.06E-47 | 1 |
|  |  |  |  |  |  |  |
| MFAP51 | 9.72E-152 | 1.587399 | 1 | 0.693 | 1.94E-148 | 2 |
| IGFBP61 | 1.96E-145 | 1.438292 | 1 | 0.956 | 3.93E-142 | 2 |
| OAF1 | 4.00E-117 | 1.234542 | 0.984 | 0.774 | 7.99E-114 | 2 |
| SEMA3C | 2.00E-109 | 1.167549 | 0.991 | 0.923 | 3.99E-106 | 2 |
| HTRA3 | 7.63E-100 | 1.38955 | 0.904 | 0.559 | 1.53E-96 | 2 |
| S100A41 | 4.85E-97 | 0.984243 | 1 | 0.944 | 9.71E-94 | 2 |
| SCARA5 | 6.73E-95 | 1.211253 | 0.897 | 0.53 | 1.35E-91 | 2 |
| TNXB | 1.43E-92 | 0.983742 | 0.89 | 0.564 | 2.87E-89 | 2 |
| JUN | 1.51E-91 | 0.763472 | 1 | 1 | 3.03E-88 | 2 |
| AHNAK | 2.68E-91 | 0.757715 | 0.998 | 0.943 | 5.36E-88 | 2 |
|  |  |  |  |  |  |  |
| TIMP31 | 4.33E-106 | 1.470376 | 1 | 0.956 | 8.66E-103 | 3 |
| CYP1B11 | 1.00E-97 | 1.202913 | 1 | 0.975 | 2.00E-94 | 3 |
| ANTXR1 | 4.13E-72 | 0.918153 | 0.958 | 0.849 | 8.26E-69 | 3 |
| IGFBP7 | 4.58E-68 | 0.953461 | 0.958 | 0.756 | 9.16E-65 | 3 |
| RHOBTB3 | 2.38E-65 | 0.952775 | 0.934 | 0.826 | 4.75E-62 | 3 |
| LUM | 2.81E-60 | 0.894319 | 0.984 | 0.954 | 5.61E-57 | 3 |
| IGFBP51 | 1.77E-56 | 1.610026 | 0.992 | 0.943 | 3.54E-53 | 3 |
| GALNT1 | 1.09E-46 | 0.756113 | 0.926 | 0.849 | 2.18E-43 | 3 |
| NPR3 | 5.79E-46 | 0.668391 | 0.639 | 0.347 | 1.16E-42 | 3 |
| HEG1 | 1.84E-45 | 0.682609 | 0.952 | 0.871 | 3.68E-42 | 3 |
|  |  |  |  |  |  |  |
| IGFBP71 | 1.08E-144 | 1.718618 | 0.989 | 0.752 | 2.16E-141 | 4 |
| RPS26 | 1.57E-137 | 0.923247 | 1 | 0.998 | 3.13E-134 | 4 |
| IFI271 | 1.32E-126 | 1.559809 | 0.96 | 0.633 | 2.64E-123 | 4 |
| NUPR11 | 3.41E-112 | 1.155056 | 1 | 0.993 | 6.82E-109 | 4 |
| FTH1 | 1.35E-108 | 0.781483 | 1 | 1 | 2.71E-105 | 4 |
| DRAM1 | 3.31E-77 | 0.908457 | 0.918 | 0.763 | 6.62E-74 | 4 |
| ITGB81 | 7.10E-77 | 1.135545 | 0.79 | 0.536 | 1.42E-73 | 4 |
| XIST1 | 3.04E-75 | 0.878127 | 0.838 | 0.54 | 6.09E-72 | 4 |
| CEMIP | 7.92E-69 | 1.292079 | 0.912 | 0.68 | 1.58E-65 | 4 |
| TXN | 1.97E-62 | 0.788704 | 1 | 0.997 | 3.95E-59 | 4 |
|  |  |  |  |  |  |  |
| IGFBP31 | 3.61E-83 | 2.295708 | 0.982 | 0.922 | 7.22E-80 | 5 |
| TPD52L1 | 1.95E-80 | 1.55523 | 0.875 | 0.617 | 3.90E-77 | 5 |
| RPS261 | 4.80E-76 | 0.721956 | 1 | 0.998 | 9.59E-73 | 5 |
| NRN1 | 5.03E-74 | 0.98662 | 0.75 | 0.397 | 1.01E-70 | 5 |
| LUM2 | 8.33E-70 | 1.10445 | 1 | 0.953 | 1.67E-66 | 5 |
| XIST2 | 1.59E-69 | 0.804418 | 0.918 | 0.542 | 3.18E-66 | 5 |
| MEST | 8.65E-64 | 1.906637 | 0.754 | 0.449 | 1.73E-60 | 5 |
| ANTXR12 | 3.83E-53 | 0.864293 | 0.954 | 0.853 | 7.66E-50 | 5 |
| PABPC1 | 3.20E-45 | 0.763357 | 1 | 0.993 | 6.40E-42 | 5 |
| ERRFI11 | 5.77E-45 | 1.00776 | 0.925 | 0.808 | 1.15E-41 | 5 |
|  |  |  |  |  |  |  |
| CLEC7A | 0 | 0.999373 | 0.587 | 0.001 | 0 | 6 |
| LILRB1 | 1.75E-283 | 0.43833 | 0.478 | 0.001 | 3.49E-280 | 6 |
| LINC01857 | 3.55E-197 | 0.383269 | 0.304 | 0 | 7.11E-194 | 6 |
| DOK2 | 4.21E-189 | 0.362941 | 0.348 | 0.001 | 8.41E-186 | 6 |
| FPR3 | 5.13E-156 | 0.270529 | 0.261 | 0 | 1.03E-152 | 6 |
| SNCA | 1.15E-143 | 0.484551 | 0.435 | 0.006 | 2.30E-140 | 6 |
| HCK | 7.52E-127 | 0.680654 | 0.609 | 0.001 | 1.50E-123 | 6 |
| PTAFR | 2.64E-116 | 0.657786 | 0.522 | 0.009 | 5.29E-113 | 6 |
| PDGFB | 4.49E-112 | 0.563606 | 0.413 | 0.009 | 8.99E-109 | 6 |
| SDS | 6.15E-112 | 1.903586 | 0.413 | 0.009 | 1.23E-108 | 6 |
